# A Life-Course Perspective on Older Workers in Workplaces Undergoing Transformative Digitalization

**DOI:** 10.1093/geront/gnac181

**Published:** 2022-12-12

**Authors:** Kathrin Komp-Leukkunen

**Affiliations:** Faculty of Social Sciences, University of Helsinki, Helsinki, Finland

**Keywords:** Life-course perspective, Retirement, Technology, Work

## Abstract

Workplaces are digitalizing, which leaves many older individuals with the choice between upskilling and early retirement. How they approach this choice influences their financial well-being, the size of the workforce, and the financial sustainability of pension schemes. The present article explores how the life-course perspective can be used to explore the situation of older workers in workplaces undergoing transformative digitalization. The life-course perspective suggests that the transformative digitalization of workplaces does not change what life events older workers encounter. However, it modifies how the events affect older workers and their capabilities for striking a work-life balance. Additionally, digitalization changes life-course structures. It can lead to earlier or later retirement, which changes the length of the life phases of middle age and old age—and possibly creates new social inequalities in life courses. The effects of transformative digitalization on older workers vary across cohorts and countries, which is typical for the principle of anchoring life courses in time and place. Future research can use the present article as a guideline for which concepts may be useful in studies on older workers in digitalizing workplaces.

Population aging is a global trend that reduces the size of the workforce and challenges the financial basis of pension schemes. Policymakers and researchers suggest reacting to these changes by extending working lives (Phillipson, 2019). Yet, longer working lives are challenging to implement—and the situation becomes even more challenging in times of digitalizing workplaces. Digitalization denotes the increasing use of “electronic tools, automatic systems, technological devices, and resources that generate, process, or store information in the form of binary code” (Sheng et al., 2022, p. 198). In workplaces, it leads, for example, to an increasing use of computers that change which and how work tasks are carried out. Numerous studies stress that older workers may be less skilled in using digital technologies, which can lower their employability and lead them to retire earlier. Such a development would clash with the attempts to extend working lives (Behaghel et al., 2014; Hudomiet & Willis, 2021). However, some studies stress that digital technologies also have the potential to support older workers. For example, these technologies may reduce the physical job demands and help older workers to better manage their health. As a result, digital technologies may support older individuals and work and help them to extend their working lives (Damman, 2016; Nagarajan & Sixsmith, 2021). Considering these potential affects, more research is needed to observe and better understand the situation of older workers in digitalizing workplaces.

Unfortunately, research on older workers in digitalizing workplaces is still in its early stages. Komp-Leukkunen et al. (2022) conducted a systematic literature review of this field and found that by late 2021, only 42 scientific articles had studied this topic in-depth. A considerable number of the studies focused on the older workers’ training needs and opportunities, with other popular topics being productivity, work participation, and labor market structures. Overall, the studies had a thin theoretical and conceptual foundation, with only one study (Marshall, 2011) suggesting a possible conceptual framework. Marshall suggests using the life-course perspective, and Komp-Leukkunen et al. (2022) echo this call. The present article discusses how concepts of the life-course perspective can be used to structure explorations of older workers in workplaces undergoing transformative digitalization.

Life-course research explains how human lives develop over time. It is often used to capture how older individuals transition through their last years of work into retirement. These studies commonly understand older workers as individuals aged 50 years or older (e.g., Madero-Cabib et al., 2021; Ogg & Renaut, 2019). Already the classical life-course studies demonstrate that life-courses depend on their social environment. Bronfenbrenner (1979) underlines that a child’s development depends on its narrower and wider social environment. Elder (1974) demonstrates that life-courses change when societies change, using the Great Depression of the 1930s as an example. The digitalization of work constitutes another such social change that can be explored with a life-course perspective (Marshall, 2011).

The present article utilizes the insight from life-course research to explore digitalization-induced transformations of work. Previous research documented that digitalization can have three kinds of effects on work: transformation, replacement, and creation. Digital technologies can transform work tasks by giving workers new means for completing their tasks. These changes happen, for example, when computers or the internet are utilized at the workplace (Fossen & Sorgner, 2021; Sharit et al., 2009; Soja & Soja, 2020). Digital technologies can also replace workers, which happens especially in routine tasks that can be automated (Kurer & Gallego, 2019). Production robots and artificial intelligence are often used for this purpose (Alcover et al., 2021; Battisti & Gravina, 2021). Finally, digital technologies can create new workplaces, especially in the Information- and Communication Technology sector (Brook, 2009; Marshall, 2011). The individuals in these workplaces are dealing with the entire range of digital technologies, ranging from computers to virtual realities. This article concentrates on transformative digitalization to be able to apply the life-course perspective to a more defined and homogenous phenomenon. This approach facilitates the clarity of analysis and argumentation.

On the following pages, this article discusses the key concepts in life-course research and then explores how they frame our extant knowledge on older workers in workplaces undergoing transformative digitalization. These key concepts are the ones relating to critical points of time (life events, the origins of life-course effects, and turning points), capturing periods in our lives (life phases and life-course structures), and explaining country differences (lives in time and place). Subsequently, recommendations for future research on older workers in digitalizing workplaces are formulated.

## Life Events, the Origins of Life-Course Effects, and Turning Points

The life-course perspective helps us better understand human lives by identifying which events in a person’s life are the most important ones. These most important events act as signposts. They give life courses a structure, and they can steer lives into new directions. The central concept used is that of the *life event*, which is an event that a person considers to be of exceptional importance in their own life (Komp-Leukkunen, 2020). Events that are often considered life events are, for example, weddings, the birth of children, and retirement. Life events can trigger long-term effects, which are called *life-course effects* (Grenier, 2012). Such life-course effects can, for example, be frugal behavior that is caused by a considerable financial loss (Gray & Dagg, 2019). If a life event steers the course of a person’s life into a new direction, then this life event is also considered a *turning point*. For example, a health scare can act as a turning point if it motivates an individual who is not health-oriented to adopt a healthy lifestyle (Grenier, 2012). An important characteristic of these key concepts is that they are subjective. There are no definitive lists of what constitutes a life event, causes a life-course effect, or acts as a turning point. Instead, these concepts have to be understood from the perspective of the individuals concerned (Komp-Leukkunen, 2020).

Previous research clearly showed that transformative digitalization of the workplace does not constitute a life event, the origin of a life-course effect, or a turning point for older individuals. Instead, it constitutes a change in the workplace environment (Oksa et al., 2021; Soja & Soja, 2020; Van Yperen & Wörtler, 2017). Moreover, workplaces may not be the first places where older individuals come into contact with digital technologies. Many older individuals use digital technologies in their private lives, which gives them experiential knowledge that they can transfer to their workplaces (Gilleard & Higgs, 2008). However, several studies showed that transformative digitalization of workplaces can modify life events, the life-course effects they cause, and turning points. Such modifications occur when it comes to the onset of health problems, changes in the family network, and retirement (Damman, 2016; Nagarajan & Sixsmith, 2021; Zhan, 2016).

The onset of health problems can constitute a life event for older workers. It can lead them to restructure their daily activities, reset priorities, and consider early retirement (Grenier, 2012; Maresova et al., 2019). Thus, the onset of health problems can trigger life-course effects for older workers, and it can act as a turning point in their life courses. Several studies suggest that the digitalization of workplaces may help older workers better manage their health. Digital solutions may ease the physical demands of work tasks, for example, by rendering the carrying of paper files and folders obsolete or by using sensors to detect and reduce workplace hazards (Sheng et al., 2022). Digital solutions can prevent health problems among older workers, and they can make previously difficult tasks feasible for older workers with health problems (Hudomiet & Willis, 2021). Likewise, digital solutions for remote work may allow older individuals with health problems to work from home, which can help them better manage their health (Damman, 2016). On the flipside, the digitalization of workplaces may also create health challenges for older individuals. Older workers experience particularly high levels of anxiety and stress when using digital technologies (Tams & Hill, 2016). Remote work blurs the boundaries between work and private life, making it harder for them to switch off the technostress (Dropkin et al., 2016). Consequently, the risk for stress-related illnesses, such as burnouts, increases. Thus, transformative digitalization of workplaces may change the *timing of the life event* of the onset of health problems. Moreover, it can modify the effects of the onset of health problems, which *modifies the life-course effects* of this life event and *the likelihood that this life event will become a turning point.*

Changes in the family network may likewise constitute life events for older workers (Grenier, 2012). Older workers may start to provide care to frail kin, such as spouses and in-laws. The onset of such caregiving duties can force older workers into early retirement (Alpass et al., 2017; Damman, 2016). Thus, it can constitute a life event for older workers, and it may have life-course effects and act as a turning point. Digital technologies facilitate a home office, which can help older workers to better coordinate the timing of their work duties and caregiving obligations, which reduces work-family conflicts. Yet, carrying both activities out at home can interfere with both activities, which increases work-family conflicts (Damman, 2016; Sharit et al., 2009; Van Yperen & Wörtler, 2017; Zhan, 2016). If kin die, then the older workers may turn to their networks for emotional and social support. Digital technologies may facilitate such support over geographical distances, for example, via teleconferencing software (Baldassar & Wilding, 2020). Likewise, older workers may utilize teleconferencing software to maintain social contact when they become grandparents or when their children move out. The software reduces the travel time needed for remaining in contact with kin (Sheng et al., 2022). Thereby, it makes it easier for older workers to combine work and family demands. Thus, digital technologies do not change the *life events*. However, they may modify how older individuals deal with these events, thereby *modifying the life-course effects and the likelihood of a turning point emerging*.

Because policymakers are currently trying to delay retirement through pension reforms, the interest of researchers and policymakers in effects of workplace digitalization on retirement is particularly high (Phillipson, 2019). However, the research could not yet establish what the net effect of digitalization is. Moreover, it could not yet establish which social inequalities exist in this effect. On the one hand, several studies showed that workplace digitalization may render the skills of older workers obsolete, making them more likely to retire early (e.g., Behaghel et al., 2014; Greenan & Messe, 2018; Hudomiet & Willis, 2021). Such a development is particularly likely when older workers are not included in workplace training (Lee et al., 2008; Magnani, 2006; Staufer, 1992; Van Dalen et al., 2015). On the other hand, Lee et al. (2020) suggest that digital technologies can offset age-related productivity declines, which can keep older workers in the workforce until an older age. Sheng et al. (2022) argue that instructions for using digital technologies may be more widely available than instructions for some company-internal processes. Therefore, older workers may find self-learning easier when dealing with digital technologies, which increases their employability and keeps them in the workforce until a later age. Zhan (2016) and Van Yperen and Wörtler (2017) add that digital technologies may make paid work so accessible to older individuals that these individuals increasingly stream into bridge employment, meaning paid work after retirement. Such a step would weaken the synchronicity between the end of paid work and the start of receiving old age pensions. Also, it would raise questions about how retirement should be defined. Thus, transformative digitalization of workplaces may *change the timing of the life event* of retirement, it may create *new social inequalities in the timing of this life event,* and it may *change what the life event of retirement looks like*.

## Life Phases and Life-Course Structures

Life-course research uses life phases and life-course structures to portray life courses in broad strokes. *Life phases* are homogenous periods in a person’s life (Komp-Leukkunen, 2020). Individuals usually engage in a characteristic activity throughout a life phase, such as attending educational classes or working for pay. Moreover, life phases often start and end with a life event, such as entering and leaving the labor market (Bernardi et al., 2019; Kohli, 2007). *Life-course structures* string several life phases together to obtain a simplified representation of the life course (Macmillan, 2005). The most commonly used life-course structure is the tripartite one that describes life courses as a sequence of youth, middle age, and old age. Youth is dedicated to education, middle age to paid work, and old age to retirement (Kohli, 2007; Komp-Leukkunen, 2020).

The effects of the transformative digitalization of workplaces on retirement affects the life phase of old age. Retirement is often used as a marker for the onset of old age (Kohli, 2007; Komp-Leukkunen, 2020). Studies showed that work digitalization may lead to earlier or later retirement (Behaghel et al., 2014; Greenan & Messe, 2018; Hudomiet & Willis, 2021; Lee et al., 2020). Consequently, the *life phase of old age may start earlier or later, making it longer or shorter*. Moreover, the suggestion that digital technologies may increase workforce participation after retirement (Van Yperen & Wörtler, 2017; Zhan, 2016) raises questions about *how the life phase of old age should be defined*. In this situation, defining it as the time after retirement would mean something different than defining it as the time after workforce participation.

## Lives in Time and Place

Life-course research repeatedly showed that lives vary over time and differ across countries (e.g., Zagel & Van Winkle, 2022; Zimmermann & Konietzka, 2018). The reason is that social institutions, which shape the life course, also vary over time and differ across countries. Examples of such social institutions are family structures, workplaces, and retirement regulations (Kohli, 2007). Bronfenbrenner’s (1979) ecological model stresses that lives progress in step with their social environment, from the very concrete level of our family members to the more abstract level of society as a whole. Life-course research uses the concept of *lives in time and place* to reflect this circumstance, stressing that life-course structures are typical for their historical times and geographical location (Settersten, 2018).

Life courses vary across historical time because they reflect the social change around them. As a result, differences across cohorts or even generations emerge. Cohorts are groups of individuals that are born around the same time. Generations are cohorts that possess a shared identity (Mannheim, 1928). The relevance of cohort differences for how older workers fare in digitalizing workplaces is immediately obvious. Cohorts differ in how old its members were when the process of digitalization set in. The cohort of today’s older workers experienced this process as a change in the end of their working lives, forcing them to pick up new skills at a higher age. In contrast, the cohort of today’s youths, which are tomorrow’s older workers, acquire digital skills early on in their lives (Gilleard & Higgs, 2008). The use of digital technologies is more self-evident for them. Some researchers even go so far as to suggest that digital skills constitute the shared identity that turns younger cohorts into a generation, namely that of the Millennials (Vugec & Stjepic, 2022).

The cohort differences are further heightened through historical events. A prime example is a coronavirus disease 2019 (COVID-19) pandemic, which escalated the use of digital technologies at work. During the pandemic years, older workers had little choice but to adopt transformative technologies to participate in home office-based work arrangements (Pit et al., 2021). Further historical shifts can be expected in times of high unemployment rates and in recessions, where older workers with lacking digital skills may be more likely than before to be laid off (Komp, 2018). In contrast, older workers with lacking digital skills may be less likely to be laid off in a time of low unemployment rates and in economic upturns. These mechanisms clearly show that the *impact of a transformation digitalization of workplaces on life-courses differs across cohorts*.

Life-courses differ across countries because they reflect the social structures surrounding them. Lee et al. (2020) argue that population aging drives digital training for older workers because the productivity-increases from training can offset the productivity-decline from population aging. They find that the labor productivity of more and less educated older Japanese workers increases when workplaces digitalize, but that this mechanism only occurs among less educated older Korean workers. Van Dalen et al. (2009) add that employers support older workers in acquiring digital skills, particularly when unemployment rates are low, hoping to thereby retain a sufficient number of employees in their companies. They find that in 2005, one third of British, one fourth of Greek and Spanish, and less than one tenth of Dutch employers found that their older employees were skilled in handling new technologies. About two thirds of Greek, roughly half of Spanish and Dutch, and one third of British employers expected their aging employees to display less enthusiasm for new technologies. Nagarajan and Sixsmith (2021) suggest that stakeholders may choose different strategies for increasing workforce participation in old age, with digital technologies playing a role in only some of them. These authors find that Australian, Canadian, Danish, German, Norwegian, and U.S. American companies trained their older workers in digital technologies. Canadian, German, Swedish, and U.S. American companies used digital technologies to make their older workers’ tasks easier. Only their investigation into a Japanese company shows no activities in this area. Pit et al. (2021) explore governmental strategies for addressed older workers during the COVID-19 pandemic, with the strategies chosen reflecting the governments’ perceptions of what challenges the pandemic brings to the foreground. They find that the Austrian and Thai governments used the pandemic as a trigger to offer digital training to older workers, whereas the Australian, British, Canadian, Chinese, German, Israeli, Japanese, Nigeria, Romanian, Singaporean, South Korean, Swedish, and U.S. American governments introduced no such measures. Therefore, *country-differences in how older workers fare when their workplaces undergo transformative digitalization are evident.*

## Discussion and Conclusion

Digitalization is changing workplaces, and it leaves many older workers with the choice between upskilling and early retirement. The situation of these older workers affects not only workplace productivity but also the financial sustainability of pension schemes. The study of older workers in digitalizing workplaces is still a young research field. This article explores how the life-course perspective can guide research in this field, focusing on transformative digitalization.

The life-course perspective suggests that a transformative digitalization of workplaces does not change what life events older workers encounter, meaning what meaningful events they experience. However, it can modify how the events affect the older workers’ lives and their capability for striking a work-life balance. Transformative digitalization changes what the life event of retirement looks like, and it may also shift the timing of this life event and create new social inequalities in its timing. As a result, the structure of the life course changes. We may either see a shorter middle age, which is the time dedicated to paid work, and a longer period of old age; or we may see an extended middle age with a shorter old age. The life-course perspective reveals that the effects of a transformative digitalization of workplaces on older workers vary with time and differ across countries. The corresponding cohort- and country-differences are already now clearly visible.

This article has scientific implications. It may guide future research on older workers in digitalizing workplaces, showing how the life-course perspective can be utilized for studying the different facets of this phenomenon. This article pinpoints which concepts can be used for these studies, thereby helping researchers to tie their work to a larger body of knowledge. The advantage of the life-course perspective is that it is versatile enough to capture a wide range of aspects of human lives. It can be applied to study the lives of individuals and populations, to explore social inequalities within and between countries, and to compare situations cross-sectionally and longitudinally. Thus, it can be applied to explore, for example, how older workers adapt to workplace digitalization, who the winners and losers of digitalization are, and whether younger generations are faring better than older ones.

Overall, the situation of older workers is changing considerably as workplace digitalization progresses. Yet, research on these changes is still emerging. The life-course perspective can guide future research on this topic, providing concepts to be used in the analyses. Thereby, it allows researchers to draw on a more extensive body of scientific knowledge, which can help them in their choice of research questions and methods, and in their interpretation of the findings.
